# T follicular helper cells: a potential therapeutic target in follicular lymphoma

**DOI:** 10.18632/oncotarget.22788

**Published:** 2017-11-30

**Authors:** Jordi Ochando, Mounia S. Braza

**Affiliations:** ^1^ Immunology Institute, Department of Oncological Sciences, Icahn School of Medicine at Mount Sinai, New York, NY 10029, USA

**Keywords:** immunotherapy, helper T cells, TFH cells, B cell non-Hodgkin lymphoma, follicular lymphoma

## Abstract

Follicular lymphoma (FL), the most common indolent B-cell non-Hodgkin lymphoma (B-NHL), is a germinal center (GC)-derived lymphoma. The mechanisms underlying B-cell differentiation/maturation in GCs could be also involved in their malignant transformation. Moreover, the non-malignant cell composition and architecture of the tumor microenvironment can influence FL development and outcome. Here, we review recent research advances on CD4 helper T cells in FL that highlight the pivotal role of T follicular helper (TFH) cells in a complex multicellular system where they interact with B cells during GC dynamics. After describing the mechanism of FL lymphomagenesis, we discuss the emerging evidence about TFH cell enrichment and involvement in FL tumorigenesis and in B-T cell interaction, TFH regulation by T follicular regulatory cells (TFR) and its potential effect on FL. Then, we provide an overview on the flexible interplay between the different CD4 T-cell subtypes and how this may be predicted in normal and pathologic contexts, according to the cell epigenetic state. Finally, we highlight the importance of targeting TFH cells in the clinic, summarize the main outstanding questions about TFH and TFR cells in FL, and describe strategies to potentiate FL therapy by taking into account TFH cells.

## INTRODUCTION

Most B-cell non-Hodgkin lymphomas (B-NHLs) originate from GC B cells and are associated with B-cell deregulation [1–4]. Follicular lymphoma (FL) is the second most common form of B-NHLs. FL gene expression profiling has revealed that the molecular characteristics of non-malignant tumor–infiltrating immune cells have a major influence on patient survival [5, 6]. However, the role of CD4^+^ helper T cells, and particularly of T follicular helper (TFH) cells, in FL is still unclear [7–11]. TFH cells are the specialized helper T cells in the germinal center (GC), and their differentiation is controlled by *BCL6* [12–14]. They contribute to GC formation and maintenance, to GC B-cell differentiation into plasma cells and to immunological memory. However, TFH cells also have an important role in human pathology. Indeed, their deregulation can lead to various diseases, such as autoimmune diseases, immunodeficiency disorders, and lymphoma [15–17]. Increasing evidence shows a link between TFH cells and FL [8, 11, 18–20]. Their constant crosstalk with B cells and their increased count in FL suggest that they might represent a novel therapeutic target in FL. Treatment with rituximab alone, or in combination with chemotherapy or radiotherapy, has markedly improved the overall survival of patients with advanced-stage FL. Moreover, more aggressive treatment approaches with high-dose chemotherapy and stem cell transplantation can be proposed to patients with more resistant disease, but with good performance status [21–25]. Nevertheless, the persistence of TFH cells following rituximab treatment and their enrichment in FL [26] highlight the need to precisely control TFH cell function and production in this cancer. In this review we will discuss recent findings on helper T cells in FL pathology and their prognostic significance by positioning TFH cells as the main CD4^+^ T helper cells that infiltrate FL, and the recently identified T follicular regulatory (TFR) cells as their regulator in GC responses. Specifically, we describe the intriguing interplay between TFH and TFR cells, their finely modulated interaction and spectacular plasticity with other helper T cells. We then discuss possible future research directions on TFH cells. Finally, we raise outstanding questions that need to be answered to understand how modulation of TFH cell function could improve FL therapeutic options.

### FL pathogenesis

**Figure 1:**
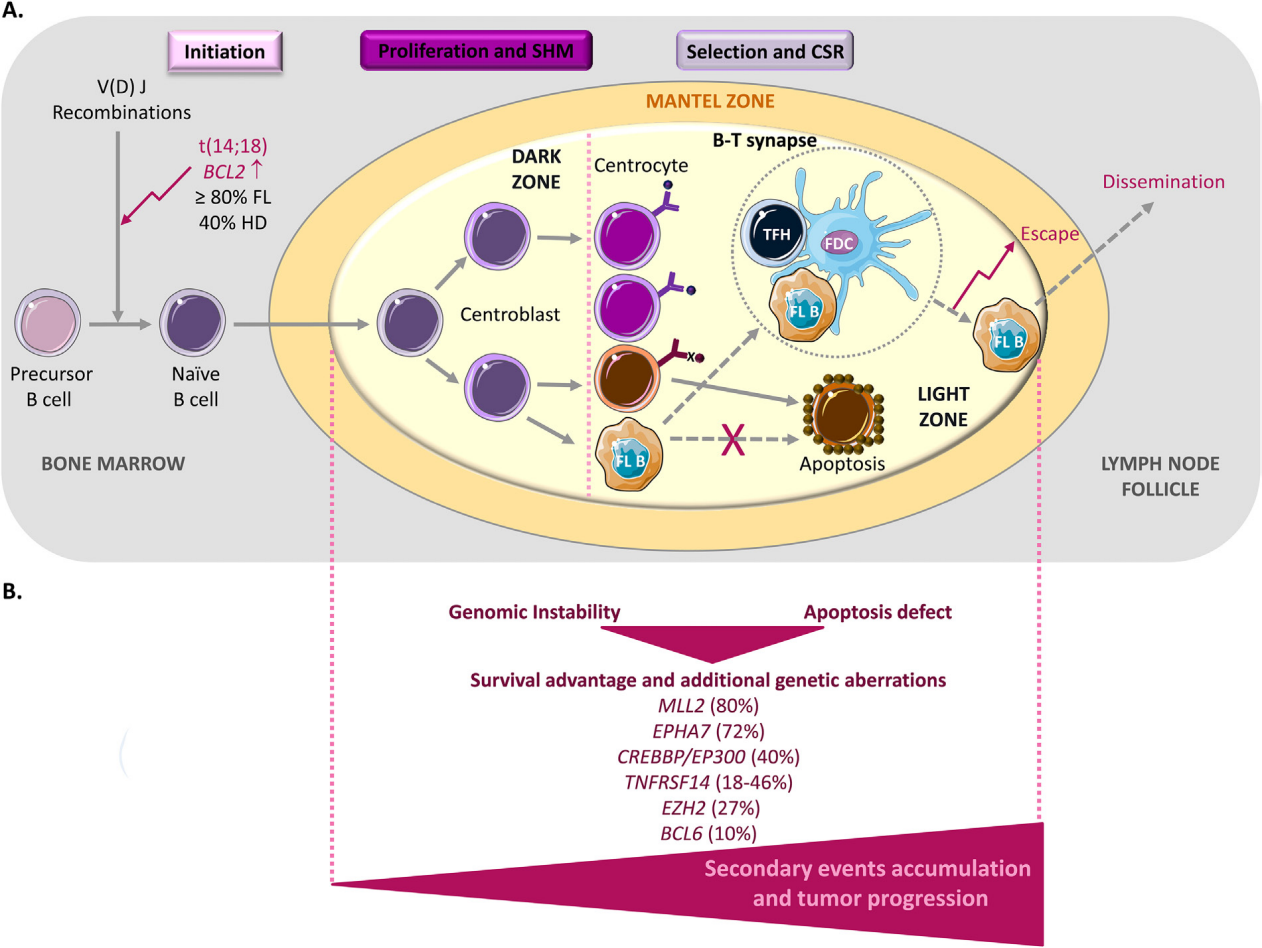
FL lymphomagenesis **(A)** The germinal center (GC) is the main source of mature B cells that produce high-affinity antibodies. In a physiological context, during the initiation step, precursor B cells that have successfully undergone V(D)J recombination and that express functional B-cell receptors become naive B cells and migrate to the T-cell zone (a T cell-rich area in lymphoid organs), where they will be fully activated as a result of their interaction with CD4^+^ T cells and antigen presenting cells (APCs). Then, they differentiate into centroblasts that undergo clonal expansion in the GC dark zone. During this intense proliferation step, somatic hypermutation (SHM) allows the diversification of the B-cell receptor repertoire. They then differentiate into centrocytes and move to the light zone, where they improve antigen binding with the help of CD4^+^ T cells (specifically, T follicular helper (TFH) cells) and follicular dendritic cells (FDCs). Centrocytes that produce an unfavorable antibody are eliminated by apoptosis (cells in brown), whereas successful centrocytes undergo immunoglobulin class-switch recombination (CSR), a biological mechanism whereby their immunoglobulin production is changed. The GC reaction finally generates memory B cells and plasma cells. In follicular lymphoma (FL), during the early initiation step, a t(14;18) chromosomal translocation occurs that involves the immunoglobulin IgH locus and the proto-oncogene *BCL2*. This leads to *BCL2* overexpression in tumor B cells (FL B) that later activate an anti-apoptotic program to promote their survival (dashed arrows). However, the finding that this translocation is also observed at low frequency in normal B cells indicates that the *BCL2* genetic alteration is necessary, but not sufficient to cause FL. **(B)** Accumulation of secondary events during the GC reaction is necessary for tumor progression. All these alterations will accumulate during somatic hypermutation (SHM) and class-switch recombination (CSR) and will contribute to dysfunctional B-T cell interactions (illustrated within the dashed circle; see Figure 2 for more details) that favor tumor growth, escape and dissemination.

Germinal centers (GC) are sites within lymph nodes where mature B lymphocytes rapidly proliferate and differentiate to produce plasma cells that secrete high-affinity antibodies during the humoral immune response to protect against infections [27]. This process includes somatic hypermutation (SHM) of the genes encoding the immunoglobulin variable region (IgV) [28–30] and class switch recombination (CSR) (Figure 1A). During their rapid division, B cells are called centroblasts, and after they have stopped proliferating and started selection, they are known as centrocytes [31, 32]. Thus, GCs are an important part of the B cell-based humoral immune response. However, the beneficial role of GC B cells in immunity is somewhat weakened by their genomic instability than can lead to lymphomagenesis. Indeed, most B-cell lymphomas originate from GC B cells [1–3]. The best examples are B-NHLs, a large group of lymphomas that includes also FL. Each B-NHL subtype is characterized by distinct genetic alterations that are often major determinants of their phenotype. About 90% of patients with FL harbor the t(14;18) translocation in which the immunoglobulin heavy chain (*IGH*) enhancer region and the B-cell lymphoma 2 (*BCL2*) gene are juxtaposed [33]. This translocation occurs early during B cell development, but contributes to lymphomagenesis at later stages during the GC reaction, possibly as a consequence of antigen stimulation (Figure 1A). The juxtaposition of the *IGH* regulatory regions leads to *BCL2* ectopic expression and activation of the anti-apoptotic program [34, 35]. However, the detection of *BCL2* genetic aberration at low frequency (40%) also in healthy individuals is one of the many evidences supporting the hypothesis that this translocation is necessary, but not sufficient for FL and that other events are required for tumor progression [36–38]. Moreover, *BCL2* deregulation provides a survival advantage that might favor the acquisition of additional genetic aberrations during repeated transits of *BCL2*-overexpressing B cells through the GC [39].

Among the secondary alterations frequently observed in FL (Figure 1B), the genetic inactivation of the histone methyltransferase *MLL2* (*KMT2B*) (in ∼89% of cases) seems to be an early event that leads to the deregulation of the B cell transcriptional program. Moreover, mutations in the genes encoding other chromatin remodelers, such as *EZH2* (in ∼27% of cases) and the acetyltransferases CREB-binding protein (*CREBBP*) and E1A-binding protein P300 (*EP300*) (in ∼40% of cases), are often observed in FL [40–44]. Rearrangements of the transcriptional repressor *BCL6* also are detected in 10% of FL [36, 45], suggesting that *BCL6* physiological function (prevention of premature activation and differentiation of GC B cells) could be diverted during B-cell malignant transformation [4]. *BCL6* targets *BCL2, TP53* and *BLIMP1* that are involved in key pathways (apoptosis, cell cycle and B-cell activation and differentiation) [3, 4, 34, 46–56]. Therefore, despite the low percentage of *BCL6* rearrangements in FL, its deregulation may be critical specifically for the DNA damage response, leading to increased tolerance against genetic alterations and to their accumulation [3, 4, 10, 57, 58]. Finally, a recent analysis of candidate genes in FL highlighted loss of *EPHA7* (a receptor tyrosine kinase and potential tumor suppressor) [59] and inactivating mutations in the *TNFRSF14* gene in 72% and 18-46% of cases, respectively [60, 61, 62]. As *TNFRSF14* interacts with molecules that control T cell biology (for example B- and T-lymphocyte attenuator, BTLA), this finding provides the first insight into the link between FL and its microenvironment.

### CD4^+^ T cells in FL microenvironment: friends or foes?

In 2004, Dave et al demonstrated that the molecular features of non-malignant immune cells within FL (i.e., the tumor microenvironment) at diagnosis are correlated with patient survival. Specifically, by gene expression analysis of tumor biopsy specimens from untreated patients with FL, they discovered two different signatures (immune response-1 and immune response-2) that reflect the gene expression profiles of the non-malignant immune cells that infiltrate the tumor. The immune response-1 signature is broadly enriched in T cells and is associated with good prognosis. Conversely, the second signature is enriched in monocytes/macrophages and is associated with bad prognosis [5, 6]. In the immune response-1 signature, the presence of CD8^+^ T cells (known as cytotoxic T lymphocytes, CTLs) in FL microenvironment is clearly associated with good prognosis and longer survival. Indeed, CTLs efficiently kill tumor cells upon contact, through the release of cytokines and perforin/granzyme [63–67]. However, the specific role of CD4^+^ T cells has not been fully defined. This could be partially explained by the fact that there are various CD4^+^ T cell populations with specific and also overlapping functions, such as helper T cells and regulatory T cells (Tregs). These multifunctional cells can express transcription factors that are specific of different cell subpopulations and can elicit multiple cytokine-mediated effector functions simultaneously (see below). Therefore, their function could be influenced by their microenvironment. Moreover, several studies have shown an association between the number and the histologic pattern of FL infiltration by helper CD4^+^ T cells, Tregs and TFH cells [7, 8, 11, 66, 68-70]. These two characteristics are a strong predictor of survival and risk of histological transformation to aggressive B-NHL. Specifically, a follicular pattern (i.e., predominant intrafollicular or perifollicular localization of cells in the lymph nodes) is associated with poor survival compared with a distribution pattern outside the follicle [7–9]. Finally, transcription factor analysis showed differences within FL in the distribution and anatomical localization of regulatory and helper T cell populations that express CXCR5, the receptor for the chemokine CXCL13, including a population of T helper cells known as TFH cells that play a critical role in GC reaction [8, 11, 13, 71, 72]. Moreover, it was reported that programmed death-1 (PD-1) expression in T cells located within follicles of secondary lymphoid organs, including TFH cells, is correlated with FL clinical outcome. In lymphoma, PD-1 is involved in T cell exhaustion (reduced cell differentiation, proliferation, and effector function), thus its expression can reduce the anti-tumor response of effector T cells [73–76]. Hence, there is a real need to better understand the place and prognostic value of helper T cell populations, specifically of TFH cells in this pathology.

### New non-malignant players in FL

Early work in human cell systems supported the hypothesis that *FOXP3*, a master regulator of Treg development and function, is only expressed in CD4^+^/CD25^+^ T cells with *in vitro* immunosuppressive activity [77–79]. However, later studies suggested that T cell receptor (TCR) stimulation of CD4^+^/CD25^-^/*FOXP3*^*-*^ T cells leads to the induction of *FOXP3* expression [80–82]. Moreover, a population of CD4^+^/CD25^-^/*FOXP3*^*+*^ T cells was identified at the tumor site of human B-NHLs [83–85], suggesting that chronic stimulation of T cells might alter T cell differentiation and *FOXP3* expression, independently of CD25. This could be even more likely in cancer, where inflammation is often the starting point. Taken together, these findings suggest that the antitumor response can be increased by targeting CD25^+^ Treg cells. However, the residual CD25^-^/*FOXP3*^*-*^ or *FOXP3*^*+*^ T cells that can mediate immune suppression would still remain and inhibit the host antitumor response. In this context, it is important to take into account TFH cells, a major sub-population of CD4^+^ T cells that express PD-1, but not CD25, and their main regulators TFR cells [12, 13, 85, 86].

#### TFH cells

TFH cells have been the subject of intense investigation, but their true identity remains elusive. These cells are considered to be a distinct T helper cell subset with an essential role in adaptive immunity. Specifically, they contribute to GC formation, B cell development and maturation, and immunoglobulin class switching. Several cell surface molecules, such as CXCR5, PD-1 and inducible T-cell co-stimulator (ICOS), have been used as TFH cell markers [12, 13, 87]. In addition to their anatomical location in GC, TFH cells display features of specialized helpers of B cells, such as expression of interleukin 21 (IL-21), an inducer of B cell functions (activation, proliferation and differentiation), and of co-stimulatory molecules, for instance CD40 ligand (CD40L) and ICOS [87–90].

Despite this confined functional definition, the TFH compartment complexity and involvement in cancer, and more especially in hematological malignancies, are starting to emerge [91–95].

Indeed, a recent study has found significantly higher plasma levels of TFH cells that also strongly express ICOS, PD-1, and IL-21 at pretreatment in patients with acute lymphoblastic leukemia, multiple myeloma or NHL, compared with healthy donors [96]. Moreover, comparison of TFH cell count and ICOS, PD-1, and IL-21 expression in these three groups of patients showed that they were the highest in patients with NHL. These authors also observed a negative correlation between TFH cell number and therapeutic effect, implying that TFH might have a prognostic value in the clinic [96].

**Figure 2:**
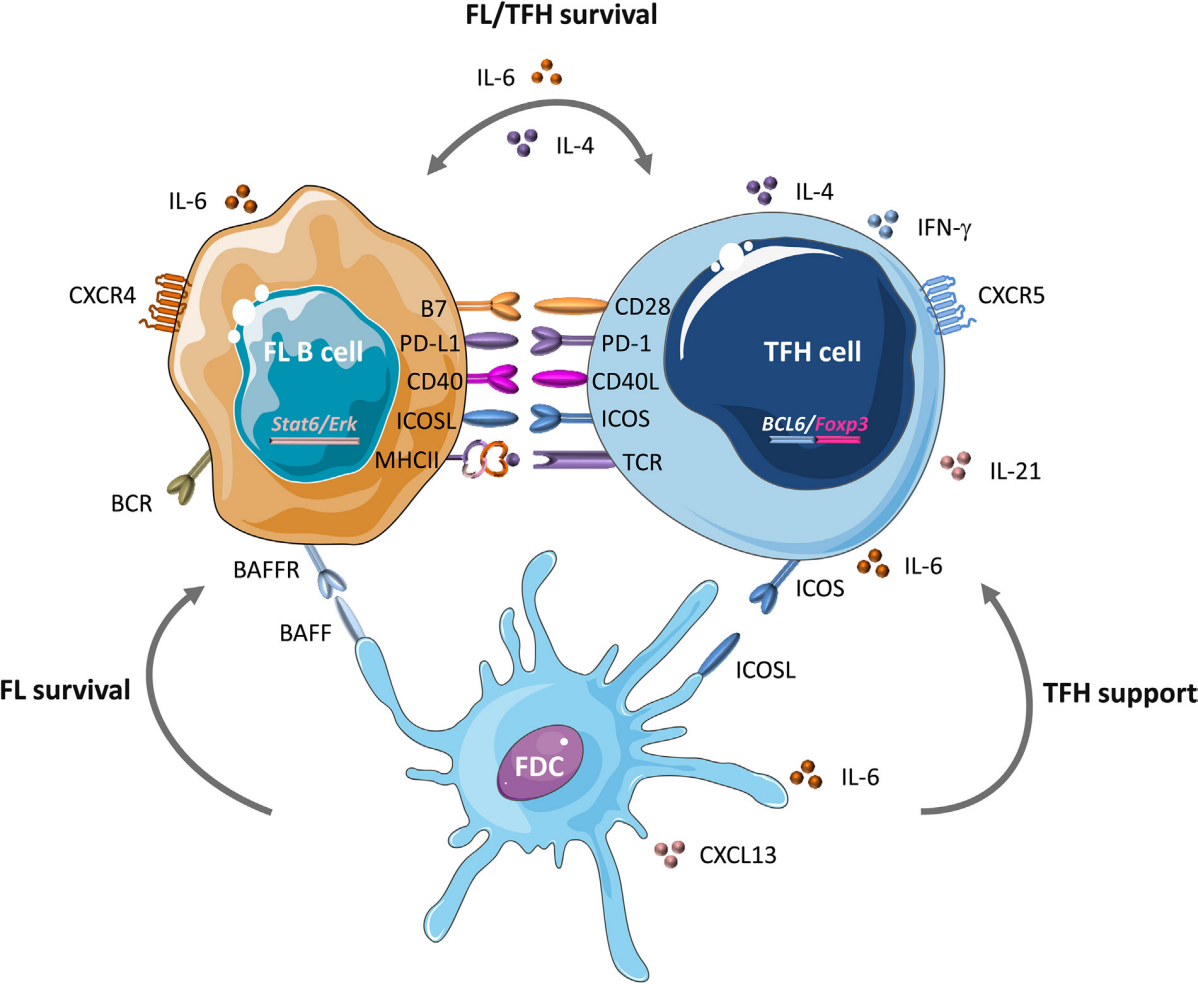
B-T synapse in FL In GCs, at the B-T cell border, TFH cells interact with different cell types and secrete several molecules and cytokines that play a critical role in lymphomagenesis. Within altered GCs, TFH cells provide a precious support to FL B cells by displaying a specific molecular signature: upregulation of IL-6, IL-21, *FOXP3* and, most importantly, IL-4 that is associated with STAT-6 and ERK-dependent FL B cell activation. Through IL-4 and CD40-L production, TFH cells might contribute to FL B cell survival. Following interaction with TFH cells, FL B cells might acquire additional pro-tumor features. Moreover, mesenchymal stromal cells (MSCs) in the FL niche support the viability of FL-infiltrating TFH cells via an IL-6-dependent mechanism. This fine interplay between FL B and TFH cells could be the consequence of *BCL6* deregulation, which mediates CD80 and PD-L1 modulation. Within this abnormal synapse, follicular dendritic cells (FDCs), as exclusive providers of both co-stimulatory signals and antigens driven to B cells, could also be subverted by tumor B cells and the tumor niche. Their ability to secrete CXCL13, BAFF, IL-6 and CD40 give them a pivotal role in TFH and FL B cell survival [159–164].

TFH cells with strong PD-1 expression (a marker correlated with FL outcome) are expanded and active in FL microenvironment (lymphomatous follicles or residual GCs) and might result in T cell inhibition and loss of effective anticancer immune function [69, 74, 97, 98]. FL-infiltrating TFH cells display a specific gene expression profile with overexpression of IL-6 and IL-21 [11, 18] (Figure 2). Moreover, high levels of IL-4, mostly produced by TFH, have been associated with Signal Transducer and Activator of Transcription protein 6 (STAT6) and ERK-dependent FL B-cell activation [13]. Through IL-4 and CD40-L production, TFH cells might contribute to FL B-cell survival [19, 20, 99]. In turn, the FL niche, which includes mesenchymal stromal cells (MSCs), supports the viability of FL-infiltrating TFH cells through an IL-6-dependent mechanism [18]. Interestingly, MSCs in the tumor microenvironment can induce *FOXP3* expression in TFH cells, thus mediating their conversion to TFR cells. This suggests that TFR can be derived from TFH cells in FL [18]. Altogether, these evidences stress the need to take into account and to control TFH cell function and production in FL.

#### TFR cells

TFR cells are also located in the GC and share phenotypic characteristics of TFH and classical Treg cells [18, 100–103]. They show a subtle and flexible balance between their opposite TFH and Treg identities, and should be considered as a separated cell subset. Although many aspects of their biology and origin are still unclear, TFR cells have been identified as a distinct subpopulation of CD4 T helper cells that express *FOXP3*, acquire a follicular phenotype and migrate into GCs, where they exert their specific regulatory role by modulating TFH number and function [101, 102]. In comparison with the classical Treg cells that express CD25 (an IL2 receptor with regulatory activity), TFR cells might downregulate CD25, but still retain their regulatory function, while upregulating genes associated with the TFH cell phenotype [85].

Concerning TFR role in FL, an excessively elevated number of follicular *FOXP3*^*+*^ Tregs that express CXCR5, a chemokine receptor with an important role in GC B cell migration, is associated with poor patient survival [18, 104]. Moreover, the proportion of these CD4^+^ T cells is higher in FL tissues than in normal lymph nodes. Differently from what observed in mice, FL TFR cells are partially derived from TFH. In addition, several findings suggest that in FL, Treg differentiation is directed toward the TFR subpopulation. Consequently, TFR cells are accumulated within the FL microenvironment (malignant lymph node) and preferentially localized and retained in malignant GCs [18, 105].

In FL, helper T cell differentiation is skewed in the direction of inflammatory and activation signals that could shape an adequate tumor microenvironment [11] in which Treg and TFH cells might functionally interact [11, 106]. Therefore, given that TFH and TFR cells are reciprocal and antagonistic regulators of GC responses, the TFH-TFR balance is critical for immune homeostasis and could influence FL biology.

#### TFH-TFR dichotomy

**Figure 3:**
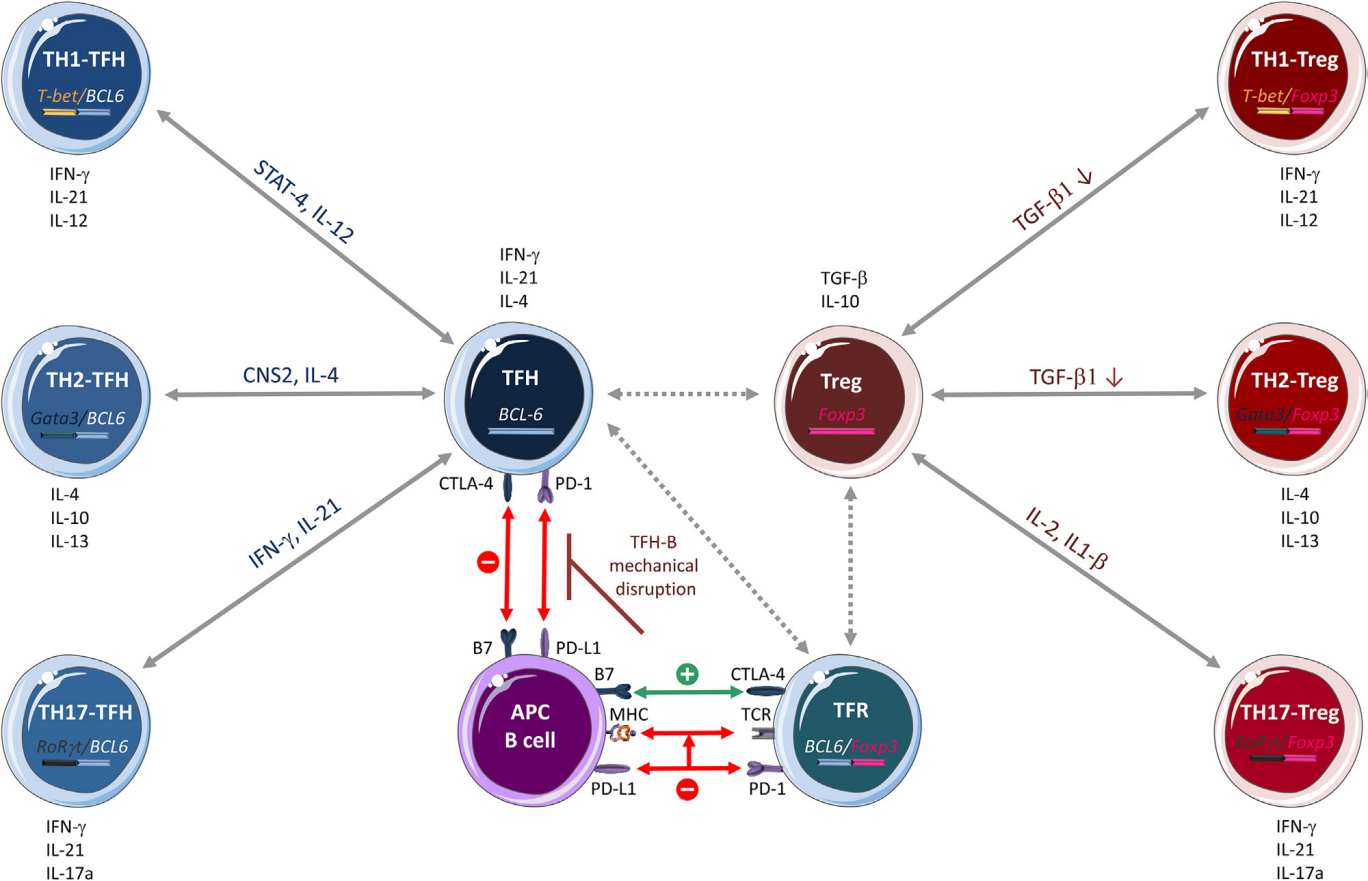
CD4^+^ T cell plasticity T helper (TH) cell subtypes and Treg lineages were previously classified simply as TH1, TH2, TH17, Treg and TFH on the basis of their specific cytokine signature (in black) and the expression of a single transcription factor. However, recent research shows that CD4^+^ T-cell subsets have an incredible inter-convertibility and trans-differentiation ability. Emerging evidence indicates that Treg and TFH cells, at least, are in the center of a flexible, interconnected system in which cells are in a multifunctional poised state and might be responsive to a multitude of cytokines and transcription factors that influence their differentiation and function. TFH cells can differentiate into TH1 and TH2 cells that express two transcription factors (*BCL6* and *T-bet* or *GATA3*, respectively) and secret specific molecules, such as STAT-4 and IL-12 (TFH/TH1 hybrid cells) and CNS2 and IL-4 (TFH/TH2 hybrid cells). Likewise, Treg cells can differentiate into TH1, TH2 and TH17 by expressing two transcription factors (*FOXP3* and *T-bet* or *GATA3* or *ROR γt*, respectively) and by modulating the expression/activity of various molecules, such as TGF-β (Treg/TH1 and Treg/TH2 hybrid cells) and IL-2 and IL-1β (Treg/TH17 hybrid cells). This fine adaptation to the microenvironment is possible through a complex epigenetic remodeling process regulated by cell-specific transcription factors. In this complex cellular system, the mechanisms of Treg and TFH cell interplay are not fully known. A recent hypothesis reported by Sage et al on the molecular interaction between TFH and T follicular regulatory (TFR) cells is illustrated here. The PD-1/CTLA-4 couple contributes to the regulation of the TFR/TFH ratio. PD-1, which is highly expressed in TFH and TFR cells, exerts an inhibitory effect on TFR cell function/differentiation and limits TFH cell function. By limiting the TCR stop signal, PD-1 increases the number of B cells that TFH cells encounter, thus preventing their too fast proliferation and allowing proper B-cell activation. CTL4-4 is highly expressed by TFR cells and negatively controls their differentiation and expansion and consequently, also TFH cell fate. The high affinity interaction of CTLA-4 on TFR cells with B7 on B cells might prevent, through mechanical disruption of TFH-B cell contacts, TFH cell binding to B cells. According to the nature of the stimulus and the microenvironment (for example, a pro-tumor context), PD-1 and CTLA-4 might modulate differently the follicular program. Green, activation; red, inhibition; dashed arrows, interactions that have not been confirmed experimentally.

The complex relationship between TFH and TFR cells is dictated by a dichotomy driven by specific signals, in which the TFR/TFH ratio is defined according to their anatomical location and the inflammatory response strength. Basically, B7 molecules and the PD-1/CTLA-4 couple are the most important factors that regulate GC dynamics through TFR/TFH modulation (Figure 3) [107, 108]. Little is known on how the PD-1/CTLA-4 couple contributes to regulating the TFR/TFH ratio. PD-1 is highly expressed by TFH and TFR cells, exerts an inhibitory signal on TFR function and differentiation, and restrains TFH function. However, in a pro-inflammatory context, PD-1 limits TCR stop signal and consequently could increase the number of B cells that TFH cells might encounter, thus preventing the quick proliferation of TFH cells, giving them time to correctly activate B cells [107, 109]. In FL, where tumor B cells alter T cells by upregulating PD-1 expression, this could easily degenerate into an uncontrollable proliferation of B cells, favoring tumor progression. CTLA-4 is highly expressed by TFR cells and negatively controls their differentiation and expansion and thus TFH cells fate. In addition, the high affinity interaction of CTLA-4 at the surface of TFR cells with B7 on B cells may prevent TFH binding to B cells through mechanical disruption of TFH-B cell contacts [100, 109, 110].

In conclusion, PD-1 and CTLA-4, through their multifaceted roles in TFH and TFR biology, may drive the predominance of the TFR or TFH cell phenotype, depending on the nature of the stimulation and the microenvironment. However, it is not known how PD-1 and CTLA-4 modulate the follicular program and most importantly how these molecules act in FL. Although it is known that TFH and TFR cells are strongly interconnected in FL, it is now important to understand how they can be specifically modulated and the possible consequences of these targeted approaches.

### The incredible plasticity of CD4^+^ T cells

It is now clear that helper T cells do not fit into a restrictive model consisting of mutually exclusive T cell lineages. For instance, the recently described conversion of T helper-17 (TH17) into T helper-2 (TH2) cells [111] highlights the flexibility of the different CD4^+^ T lymphocyte subsets. Moreover, the notion of CD4^+^ T cell plasticity has been strengthened by the *ex vivo* identification of CD4^+^ T lymphocyte hybrids that co-express transcription factors of two distinct subsets [112]. T cell fate plasticity can be advantageous for the host defense against pathogens, but can also play a determinant role in tumor development.

Several evidences highlight TFH cell flexibility and interplay with other helper T lineages. First, both TFH and TH2 cells produce IL-4 that has a major role in humoral immunity [113]. Moreover, the conserved non-coding sequence 2 (CNS2) distal IL-4 enhancer, while dispensable for TH2 cell function, plays an important role in IL-4-mediated humoral responses that are mainly regulated by TFH cells [114, 115]. This relationship was confirmed by the finding that TFH cells can develop from TH2 cells *in vivo* after helminth infection [116]. As IL-4 can also promote tumorigenesis through alteration of the tumor suppressing activity of monocytes and macrophages [117–119], the overlapping phenotypical and functional characteristics of TFH and TH2 cells might also favor the tumor niche in FL. Second, both TFH and TH1 cells produce interferon gamma (IFNγ) that has a main role in immune responses. Through STAT4, IL-12 induces a transitional stage of TFH-TH1 cells that express IL-21 and *BCL6*, promoting both phenotypes. STAT4 also induces *T-bet* expression that, together with IFNγ, represses the TFH cell phenotype and functionalities, promoting full TH1 cell differentiation [120]. Moreover, human B cells might induce prominent and stable co-expression of the TH1 and TFH cell signatures during priming and antigen recall [121]. This suggests that human B cells exploit CD4^+^ T cell plasticity to render the effector T cell response more flexible. Moreover, increasing evidence indicates that transcription factors thought to be master regulators of other helper T cell lineages can also be expressed by TFH cells [101, 121–126]. This suggests that TFH cells have the potential to express a number of different lineage-defining factors, depending on the environmental conditions (Figure 3). Therefore, it is important to determine whether in FL, tumor B cells might influence this plasticity.

Likewise, Tregs can convert into TH1, TH2 or TFH lymphocytes [127, 128] (Figure 3). Mouse *FOXP3*^*+*^ Tregs express also *T-bet*, a TH1 cell-transcription factor [129] [17, 112, 130–132]. Similarly, human memory *FOXP3*^*+*^ Tregs express also *RORγt*, the main transcription factor of TH17 cells [133], and they can convert into TH17 upon IL-2 and IL-1β stimulation, or in the presence of IL-6 and TGF-β [112, 134–136].

Many aspects of T-cell plasticity still need to be elucidated, including the identification of the expression patterns and the mechanistic distinction between stable cells and more flexible subsets within a given cell lineage during physiological immune responses and in FL. Importantly, the ability of transcription factors to redefine or to modulate cell fate depends on whether they can regulate epigenetic events in a given setting. Recent data suggest that the helper T cell lineage-defining transcription factors *T-bet, GATA3*, and *FOXP3* act in part by regulating the epigenetic environment of the cells in which they are expressed [137–141]. Along with lineage-specific transcription factors, epigenetic regulation (histone modifications and DNA methylation) plays an important role in helper T cell plasticity, and consequently in cancer initiation and development [140, 142–149]. Indeed, H3K4 methylation and H3K27 methylation are associated with permissive and repressive epigenetic states, respectively, and are two histone modifications that are important during helper T cell differentiation. In progenitor cells, the co-localization of these two histone modifications, which is called bivalent chromatin, represents an epigenetic state whereby genes are poised to be expressed, but with the potential to be turned on or off during the next cell developmental stage, depending on which signaling pathway(s) will be activated in response to environmental cues [150–152]. As a consequence, the stability of helper T cells may be challenged by abnormal environmental stimuli that lead to the expression of poised genes in a not expected cell lineage, as it may be the case in the highly deregulated tumor microenvironment. Moreover, recent reports suggest a role for DNA methylation and histone modifications in TFH-cell differentiation [153], supporting the high potential of TFH cells for reprogramming into other TH cell subsets [123, 124].

For all these reasons, it is important to elucidate the mechanisms by which each transcription factor functionally regulates gene expression both alone and in combination, as well as the global epigenetic background of each T cell lineage. This could allow predicting the consequences of their overlapping expression patterns in normal and pathogenic settings.

### Future direction of research

It is increasingly clear that some tumor-infiltrating lymphocytes may be friends, while others may be foes. Indeed, the overall increase in their number may not reflect a better prognosis, if this does not concern the right cell subsets with the right topography and at the right timeline. This may help explaining, in part, the disappointing clinical results of cancer vaccination strategies [154]. Therefore, therapies should act not only on FL B cells, but also on specific T cell populations to achieve acceptable results. For that, each subpopulation must be more precisely identified.

In the complex set of cellular interactions within the FL microenvironment, tumor-infiltrating CD4^+^ T cells display overlapping functions with critical roles. Among them, TFH cells are strongly enriched in the FL microenvironment and support malignant FL B cells, partly through CD40-L and IL-4. TFH cells are tightly regulated by and interact early with B cells in the GC. This FL-infiltrating T cell population plays a major role in FL biology and in follicular lymphomagenesis. Their precise mechanism of action and the timeline of their action need to be determined. TFR cells, which share both Treg and TFH features, exert a suppressive function on GC responses. The interplay between TFH and TFR cells has an impact on FL biology because of their reciprocal regulation. Most importantly, the persistence of TFH and TFR cells following rituximab treatment [26] shows the limit of the current therapies and is a valuable reason to strongly support the development of combinatory treatments in the clinic. Indeed, rituximab treatment leads to depletion of circulating naive B cells that express CD20, but this does not necessary reflect a B-cell deficiency in tissues [26, 155, 156], thus leaving the memory B-cell compartment intact and resistant to treatment. Moreover, pre-B and mature plasma cells do not express CD20 and are consequently not targeted by rituximab. Contrary to what reported in mice where GC B-cell depletion results in TFH cell loss [26, 157], Wallin et al showed that human TFH and TFR cells are not affected by GC B-cell depletion, suggesting that human TFH and TFR cells require B cells for their formation but not for their maintenance. Therefore, TFH and TFR subsets also should be targeted, to reduce relapse following rituximab treatment and to prevent the rapid reconstitution of the pathological GC response, once the B-cell pool begins to recover. Despite the interesting data on TFH enrichment and involvement in FL (Box 1), there is no clinical trial targeting these cells in FL yet. On the other hand, some clinical studies in the USA are assessing in patients with lupus the association of rituximab (after its unexpected failure on its own) with belimumab, an inhibitor of B-cell activating factor (BAFF, known to promote TFH cell formation) to reduce the abnormal GC activity orchestrated by TFH cells. These trials include the open-label CALIBRATE study (rituximab followed by belimumab for lupus nephritis) and the SYNBIoSe study (Synergetic B cell Immodulation in systemic lupus erythematosus). Moreover, a very recent study on the dynamic changes of peripheral TFH cells in patients with malignant lymphoid disease during treatment showed a negative correlation between TFH numeration, therapeutic effect and remission [96].

**Box 1:** Summary of what is known about TFH cells in FL

1) TFH cells are strongly enriched in FL. 2) TFH cells contribute to the FL niche by expressing IL-6, IL-21, IL-4 and CD40-L. 3) TFH and TFR cells support FL viability, partly through an IL-6 dependent mechanism. 4) In FL, TFH cells could convert to TFR cells by expressing *FOXP3*. 5) TFH-TFR balance could partly regulate FL biology. 6) Overall, there is a negative correlation between TFH numeration and the therapeutic effect in patients with malignant lymphoid disease.

Also, it remains unclear whether TFH cells can switch phenotype and the exact nature of their relationship with the other helper T cells in physiological and pathological settings. Novel therapeutic strategies that target the relevant transcription factors and epigenetic modifications may help to identify and control their mechanisms of action. The ultimate goal would be exploiting T-cell plasticity for reprogramming T-cell populations towards a specific phenotype, for instance, to reduce inflammation or improve the anti-tumor immune response.

More specifically, a better understanding of the key TFH cell markers (CD28, CD80, CXCR5…) and cytokine/chemokine signaling (IL-6, IL-21, CXCL13…) would offer a large panel of novel therapeutic options for targeting these cells. In addition, given the important role of PD-1/CTLA4 and of PD-L2/ICOS-L expression in regulating the quality, quantity and consequently the balance of TFH/TFR and B cells in GC, strategies to modulate the expression/function of these molecules could be useful in FL to reverse CTL anergy. Furthermore, as it is mostly expressed by TFH cells, PD-1 upregulation in FL microenvironment could represent a potential prognostic factor. Lastly, additional functional studies on *TNFRSF14*, a gene mutated in FL, are needed to elucidate its specific role and interaction with TFH cells via BTLA, which is highly expressed by TFH cells only in B-cell small lymphocytic lymphoma and chronic lymphocytic leukemia [158]. Preclinical studies are required to determine whether some of these molecules show possible efficacy and their safety before contemplating clinical trials.

Finally, several outstanding questions on the different T cell subtypes present in the FL microenvironment remain (Box 2). As TFH and TFR cells are both affected in FL and somehow inter-connected, understanding whether they can be interchangeable and how they are regulated would be helpful for their specific exploitation as therapeutic targets. *FOXP3* induction raises the question of whether its expression is a common activation marker of the Treg and TFR cell subsets.

**Box 2:** Outstanding questions

1) What are the limits of T helper cell plasticity in FL? 2) Should T helper hybrid populations be considered to be specialized cells with a particular function or a differentiation/conversion intermediate? 3) Could TFR cells derive from Treg and/or TFH cells? Are they inter-convertible or fully separated? 4) Which mechanisms dictate TFH/TFR dichotomy in FL? 5) Are TFH and TFR cells the only follicular T-cell subsets present in the FL niche?

Ultimately, the future seems to belong to therapeutic strategies that do not only destroy directly cancer cells, but also modify the tumor niche/environment to deprive them of the indispensable support for their survival. To this aim, it is necessary to combine molecular epigenetic and immunological studies to predict the potential of key transcription factors to over-ride the epigenetic changes imposed by the tumor. Thus, a better understanding of the early and late epigenetic events might help developing specific drugs for each cancer stage and typology.

## References

[1] Kuppers R, Klein U, Hansmann ML, Rajewsky K (1999). Cellular origin of human B-cell lymphomas. N Engl J Med.

[2] Stevenson F, Sahota S, Zhu D, Ottensmeier C, Chapman C, Oscier D, Hamblin T (1998). Insight into the origin and clonal history of B-cell tumors as revealed by analysis of immunoglobulin variable region genes. Immunol Rev.

[3] Basso K, Dalla-Favera R (2015). Germinal centres and B cell lymphomagenesis. Nat Rev Immunol.

[4] Basso K, Dalla-Favera R (2012). Roles of BCL6 in normal and transformed germinal center B cells. Immunol Rev.

[5] Dave SS, Wright G, Tan B, Rosenwald A, Gascoyne RD, Chan WC, Fisher RI, Braziel RM, Rimsza LM, Grogan TM, Miller TP, LeBlanc M, Greiner TC (2004). Prediction of survival in follicular lymphoma based on molecular features of tumor-infiltrating immune cells. N Engl J Med.

[6] Staudt LM, Dave S (2005). The biology of human lymphoid malignancies revealed by gene expression profiling. Adv Immunol.

[7] Carreras J, Lopez-Guillermo A, Fox BC, Colomo L, Martinez A, Roncador G, Montserrat E, Campo E, Banham AH (2006). High numbers of tumor-infiltrating FOXP3-positive regulatory T cells are associated with improved overall survival in follicular lymphoma. Blood.

[8] Carreras J, Lopez-Guillermo A, Roncador G, Villamor N, Colomo L, Martinez A, Hamoudi R, Howat WJ, Montserrat E, Campo E (2009). High numbers of tumor-infiltrating programmed cell death 1-positive regulatory lymphocytes are associated with improved overall survival in follicular lymphoma. J Clin Oncol.

[9] Farinha P, Al-Tourah A, Gill K, Klasa R, Connors JM, Gascoyne RD (2010). The architectural pattern of FOXP3-positive T cells in follicular lymphoma is an independent predictor of survival and histologic transformation. Blood.

[10] Yu D, Rao S, Tsai LM, Lee SK, He Y, Sutcliffe EL, Srivastava M, Linterman M, Zheng L, Simpson N, Ellyard JI, Parish IA, Ma CS (2009). The transcriptional repressor Bcl-6 directs T follicular helper cell lineage commitment. Immunity.

[11] Hilchey SP, Rosenberg AF, Hyrien O, Secor-Socha S, Cochran MR, Brady MT, Wang JC, Sanz I, Burack WR, Quataert SA, Bernstein SH (2011). Follicular lymphoma tumor-infiltrating T-helper (T(H)) cells have the same polyfunctional potential as normal nodal T(H) cells despite skewed differentiation. Blood.

[12] Crotty S (2011). Follicular helper CD4 T cells (TFH). Annu Rev Immunol.

[13] Fazilleau N, Mark L, McHeyzer-Williams LJ, McHeyzer-Williams MG (2009). Follicular helper T cells: lineage and location. Immunity.

[14] Crotty S (2014). T follicular helper cell differentiation, function, and roles in disease. Immunity.

[15] Lindqvist M, van Lunzen J, Soghoian DZ, Kuhl BD, Ranasinghe S, Kranias G, Flanders MD, Cutler S, Yudanin N, Muller MI, Davis I, Farber D, Hartjen P (2012). Expansion of HIV-specific T follicular helper cells in chronic HIV infection. J Clin Invest.

[16] Hu S, Young KH, Konoplev SN, Medeiros LJ (2012). Follicular T-cell lymphoma: a member of an emerging family of follicular helper T-cell derived T-cell lymphomas. Hum Pathol.

[17] Feng T, Cao AT, Weaver CT, Elson CO, Cong Y (2011). Interleukin-12 converts Foxp3+ regulatory T cells to interferon-gamma-producing Foxp3+ T cells that inhibit colitis. Gastroenterology.

[18] Brady MT, Hilchey SP, Hyrien O, Spence SA, Bernstein SH (2014). Mesenchymal stromal cells support the viability and differentiation of follicular lymphoma-infiltrating follicular helper T-cells. PLoS One.

[19] Pangault C, Ame-Thomas P, Ruminy P, Rossille D, Caron G, Baia M, De Vos J, Roussel M, Monvoisin C, Lamy T, Tilly H, Gaulard P, Tarte K (2010). Follicular lymphoma cell niche: identification of a preeminent IL-4-dependent T(FH)-B cell axis. Leukemia.

[20] Rawal S, Chu F, Zhang M, Park HJ, Nattamai D, Kannan S, Sharma R, Delgado D, Chou T, Lin HY, Baladandayuthapani V, Luong A, Vega F (2013). Cross talk between follicular Th cells and tumor cells in human follicular lymphoma promotes immune evasion in the tumor microenvironment. J Immunol.

[21] Marcus R, Imrie K, Solal-Celigny P, Catalano JV, Dmoszynska A, Raposo JC, Offner FC, Gomez-Codina J, Belch A, Cunningham D, Wassner-Fritsch E, Stein G (2008). Phase III study of R-CVP compared with cyclophosphamide, vincristine, and prednisone alone in patients with previously untreated advanced follicular lymphoma. J Clin Oncol.

[22] Salles G, Seymour JF, Offner F, Lopez-Guillermo A, Belada D, Xerri L, Feugier P, Bouabdallah R, Catalano JV, Brice P, Caballero D, Haioun C, Pedersen LM (2011). Rituximab maintenance for 2 years in patients with high tumour burden follicular lymphoma responding to rituximab plus chemotherapy (PRIMA): a phase 3, randomised controlled trial. Lancet.

[23] van Oers MH, Van Glabbeke M, Giurgea L, Klasa R, Marcus RE, Wolf M, Kimby E, van t Veer M, Vranovsky A, Holte H, Hagenbeek A (2010). Rituximab maintenance treatment of relapsed/resistant follicular non-Hodgkin’s lymphoma: long-term outcome of the EORTC 20981 phase III randomized intergroup study. J Clin Oncol.

[24] Vidal L, Gafter-Gvili A, Salles G, Dreyling MH, Ghielmini M, Hsu Schmitz SF, Pettengell R, Witzens-Harig M, Shpilberg O (2011). Rituximab maintenance for the treatment of patients with follicular lymphoma: an updated systematic review and meta-analysis of randomized trials. J Natl Cancer Inst.

[25] Dreyling M, Ghielmini M, Marcus R, Salles G, Vitolo U (2011). Newly diagnosed and relapsed follicular lymphoma: ESMO Clinical Practice Guidelines for diagnosis, treatment and follow-up. Ann Oncol.

[26] Wallin EF, Jolly EC, Suchanek O, Bradley JA, Espeli M, Jayne DR, Linterman MA, Smith KG (2014). Human T-follicular helper and T-follicular regulatory cell maintenance is independent of germinal centers. Blood.

[27] MacLennan IC (1994). Germinal centers. Annu Rev Immunol.

[28] MacLennan IC, Gray D (1986). Antigen-driven selection of virgin and memory B cells. Immunol Rev.

[29] Jacob J, Kelsoe G, Rajewsky K, Weiss U (1991). Intraclonal generation of antibody mutants in germinal centres. Nature.

[30] Berek C, Berger A, Apel M (1991). Maturation of the immune response in germinal centers. Cell.

[31] Shaffer AL, Rosenwald A, Hurt EM, Giltnane JM, Lam LT, Pickeral OK, Staudt LM (2001). Signatures of the immune response. Immunity.

[32] Klein U, Tu Y, Stolovitzky GA, Keller JL, Haddad J, Miljkovic V, Cattoretti G, Califano A, Dalla-Favera R (2003). Transcriptional analysis of the B cell germinal center reaction. Proc Natl Acad Sci U S A.

[33] Kuppers R (2005). Mechanisms of B-cell lymphoma pathogenesis. Nat Rev Cancer.

[34] Saito M, Novak U, Piovan E, Basso K, Sumazin P, Schneider C, Crespo M, Shen Q, Bhagat G, Califano A, Chadburn A, Pasqualucci L, Dalla-Favera R (2009). BCL6 suppression of BCL2 via Miz1 and its disruption in diffuse large B cell lymphoma. Proc Natl Acad Sci U S A.

[35] Czabotar PE, Lessene G, Strasser A, Adams JM (2014). Control of apoptosis by the BCL-2 protein family: implications for physiology and therapy. Nat Rev Mol Cell Biol.

[36] Klein U, Dalla-Favera R (2008). Germinal centres: role in B-cell physiology and malignancy. Nat Rev Immunol.

[37] Roulland S, Faroudi M, Mamessier E, Sungalee S, Salles G, Nadel B (2011). Early steps of follicular lymphoma pathogenesis. Adv Immunol.

[38] Schuler F, Dolken L, Hirt C, Kiefer T, Berg T, Fusch G, Weitmann K, Hoffmann W, Fusch C, Janz S, Rabkin CS, Dolken G (2009). Prevalence and frequency of circulating t(14;18)-MBR translocation carrying cells in healthy individuals. Int J Cancer.

[39] Sungalee S, Mamessier E, Morgado E, Gregoire E, Brohawn PZ, Morehouse CA, Jouve N, Monvoisin C, Menard C, Debroas G, Faroudi M, Mechin V, Navarro JM (2014). Germinal center reentries of BCL2-overexpressing B cells drive follicular lymphoma progression. J Clin Invest.

[40] Morin RD, Johnson NA, Severson TM, Mungall AJ, An J, Goya R, Paul JE, Boyle M, Woolcock BW, Kuchenbauer F, Yap D, Humphries RK, Griffith OL (2010). Somatic mutations altering EZH2 (Tyr641) in follicular and diffuse large B-cell lymphomas of germinal-center origin. Nat Genet.

[41] Bodor C, Grossmann V, Popov N, Okosun J, O’Riain C, Tan K, Marzec J, Araf S, Wang J, Lee AM, Clear A, Montoto S, Matthews J (2013). EZH2 mutations are frequent and represent an early event in follicular lymphoma. Blood.

[42] Morin RD, Mendez-Lago M, Mungall AJ, Goya R, Mungall KL, Corbett RD, Johnson NA, Severson TM, Chiu R, Field M, Jackman S, Krzywinski M, Scott DW (2011). Frequent mutation of histone-modifying genes in non-Hodgkin lymphoma. Nature.

[43] Pasqualucci L, Dominguez-Sola D, Chiarenza A, Fabbri G, Grunn A, Trifonov V, Kasper LH, Lerach S, Tang H, Ma J, Rossi D, Chadburn A, Murty VV (2011). Inactivating mutations of acetyltransferase genes in B-cell lymphoma. Nature.

[44] Okosun J, Bodor C, Wang J, Araf S, Yang CY, Pan C, Boller S, Cittaro D, Bozek M, Iqbal S, Matthews J, Wrench D, Marzec J (2014). Integrated genomic analysis identifies recurrent mutations and evolution patterns driving the initiation and progression of follicular lymphoma. Nat Genet.

[45] Niu H, Ye BH, Dalla-Favera R (1998). Antigen receptor signaling induces MAP kinase-mediated phosphorylation and degradation of the BCL-6 transcription factor. Genes Dev.

[46] Duan S, Cermak L, Pagan JK, Rossi M, Martinengo C, di Celle PF, Chapuy B, Shipp M, Chiarle R, Pagano M (2012). FBXO11 targets BCL6 for degradation and is inactivated in diffuse large B-cell lymphomas. Nature.

[47] Niu H, Cattoretti G, Dalla-Favera R (2003). BCL6 controls the expression of the B7-1/CD80 costimulatory receptor in germinal center B cells. J Exp Med.

[48] Phan RT, Dalla-Favera R (2004). The BCL6 proto-oncogene suppresses p53 expression in germinal-centre B cells. Nature.

[49] Phan RT, Saito M, Basso K, Niu H, Dalla-Favera R (2005). BCL6 interacts with the transcription factor Miz-1 to suppress the cyclin-dependent kinase inhibitor p21 and cell cycle arrest in germinal center B cells. Nat Immunol.

[50] Polo JM, Dell’Oso T, Ranuncolo SM, Cerchietti L, Beck D, Da Silva GF, Prive GG, Licht JD, Melnick A (2004). Specific peptide interference reveals BCL6 transcriptional and oncogenic mechanisms in B-cell lymphoma cells. Nat Med.

[51] Saito M, Gao J, Basso K, Kitagawa Y, Smith PM, Bhagat G, Pernis A, Pasqualucci L, Dalla-Favera R (2007). A signaling pathway mediating downregulation of BCL6 in germinal center B cells is blocked by BCL6 gene alterations in B cell lymphoma. Cancer Cell.

[52] Shaffer AL, Yu X, He Y, Boldrick J, Chan EP, Staudt LM (2000). BCL-6 represses genes that function in lymphocyte differentiation, inflammation, and cell cycle control. Immunity.

[53] Tunyaplin C, Shaffer AL, Angelin-Duclos CD, Yu X, Staudt LM, Calame KL (2004). Direct repression of prdm1 by Bcl-6 inhibits plasmacytic differentiation. J Immunol.

[54] Ci W, Polo JM, Cerchietti L, Shaknovich R, Wang L, Yang SN, Ye K, Farinha P, Horsman DE, Gascoyne RD, Elemento O, Melnick A (2009). The BCL6 transcriptional program features repression of multiple oncogenes in primary B cells and is deregulated in DLBCL. Blood.

[55] Basso K, Saito M, Sumazin P, Margolin AA, Wang K, Lim WK, Kitagawa Y, Schneider C, Alvarez MJ, Califano A, Dalla-Favera R (2010). Integrated biochemical and computational approach identifies BCL6 direct target genes controlling multiple pathways in normal germinal center B cells. Blood.

[56] Ding BB, Yu JJ, Yu RY, Mendez LM, Shaknovich R, Zhang Y, Cattoretti G, Ye BH (2008). Constitutively activated STAT3 promotes cell proliferation and survival in the activated B-cell subtype of diffuse large B-cell lymphomas. Blood.

[57] Johnston RJ, Poholek AC, DiToro D, Yusuf I, Eto D, Barnett B, Dent AL, Craft J, Crotty S (2009). Bcl6 and Blimp-1 are reciprocal and antagonistic regulators of T follicular helper cell differentiation. Science.

[58] Nurieva RI, Chung Y, Martinez GJ, Yang XO, Tanaka S, Matskevitch TD, Wang YH, Dong C (2009). Bcl6 mediates the development of T follicular helper cells. Science.

[59] Oricchio E, Nanjangud G, Wolfe AL, Schatz JH, Mavrakis KJ, Jiang M, Liu X, Bruno J, Heguy A, Olshen AB, Socci ND, Teruya-Feldstein J, Weis-Garcia F (2011). The Eph-receptor A7 is a soluble tumor suppressor for follicular lymphoma. Cell.

[60] Cheung KJ, Johnson NA, Affleck JG, Severson T, Steidl C, Ben-Neriah S, Schein J, Morin RD, Moore R, Shah SP, Qian H, Paul JE, Telenius A (2010). Acquired TNFRSF14 mutations in follicular lymphoma are associated with worse prognosis. Cancer Res.

[61] Launay E, Pangault C, Bertrand P, Jardin F, Lamy T, Tilly H, Tarte K, Bastard C, Fest T (2012). High rate of TNFRSF14 gene alterations related to 1p36 region in de novo follicular lymphoma and impact on prognosis. Leukemia.

[62] Kridel R, Sehn LH, Gascoyne RD (2012). Pathogenesis of follicular lymphoma. J Clin Invest.

[63] Braza MS, Caraux A, Rousset T, Lafaye de Micheaux S, Sicard H, Squiban P, Costes V, Klein B, Rossi JF (2010). gammadelta T lymphocytes count is normal and expandable in peripheral blood of patients with follicular lymphoma, whereas it is decreased in tumor lymph nodes compared with inflammatory lymph nodes. J Immunol.

[64] Braza MS, Klein B, Fiol G, Rossi JF (2011). gammadelta T-cell killing of primary follicular lymphoma cells is dramatically potentiated by GA101, a type II glycoengineered anti-CD20 monoclonal antibody. Haematologica.

[65] Yu P, Fu YX (2006). Tumor-infiltrating T lymphocytes: friends or foes?. Lab Invest.

[66] Alvaro T, Lejeune M, Salvado MT, Lopez C, Jaen J, Bosch R, Pons LE (2006). Immunohistochemical patterns of reactive microenvironment are associated with clinicobiologic behavior in follicular lymphoma patients. J Clin Oncol.

[67] Braza MS, Klein B (2012). Anti-tumour immunotherapy with Vgamma9Vdelta2 T lymphocytes: from the bench to the bedside. Br J Haematol.

[68] Relander T, Johnson NA, Farinha P, Connors JM, Sehn LH, Gascoyne RD (2010). Prognostic factors in follicular lymphoma. J Clin Oncol.

[69] Wahlin BE, Aggarwal M, Montes-Moreno S, Gonzalez LF, Roncador G, Sanchez-Verde L, Christensson B, Sander B, Kimby E (2010). A unifying microenvironment model in follicular lymphoma: outcome is predicted by programmed death-1--positive, regulatory, cytotoxic, and helper T cells and macrophages. Clin Cancer Res.

[70] Gribben JG (2010). Implications of the tumor microenvironment on survival and disease response in follicular lymphoma. Curr Opin Oncol.

[71] Marinova E, Han S, Zheng B (2007). Germinal center helper T cells are dual functional regulatory cells with suppressive activity to conventional CD4+ T cells. J Immunol.

[72] Yang ZZ, Novak AJ, Stenson MJ, Witzig TE, Ansell SM (2006). Intratumoral CD4+CD25+ regulatory T-cell-mediated suppression of infiltrating CD4+ T cells in B-cell non-Hodgkin lymphoma. Blood.

[73] Smeltzer JP, Jones JM, Ziesmer SC, Grote DM, Xiu B, Ristow KM, Yang ZZ, Nowakowski GS, Feldman AL, Cerhan JR, Novak AJ, Ansell SM (2014). Pattern of CD14+ follicular dendritic cells and PD1+ T cells independently predicts time to transformation in follicular lymphoma. Clin Cancer Res.

[74] Yang ZZ, Kim HJ, Villasboas JC, Chen YP, Price-Troska T, Jalali S, Wilson M, Novak AJ, Ansell SM (2017). Expression of LAG-3 defines exhaustion of intratumoral PD-1+ T cells and correlates with poor outcome in follicular lymphoma. Oncotarget.

[75] Gravelle P, Burroni B, Pericart S, Rossi C, Bezombes C, Tosolini M, Damotte D, Brousset P, Fournie JJ, Laurent C (2017). Mechanisms of PD-1/PD-L1 expression and prognostic relevance in non-Hodgkin lymphoma: a summary of immunohistochemical studies. Oncotarget.

[76] Laurent C, Charmpi K, Gravelle P, Tosolini M, Franchet C, Ysebaert L, Brousset P, Bidaut A, Ycart B, Fournie JJ (2015). Several immune escape patterns in non-Hodgkin’s lymphomas. Oncoimmunology.

[77] Baecher-Allan C, Brown JA, Freeman GJ, Hafler DA (2001). CD4+CD25high regulatory cells in human peripheral blood. J Immunol.

[78] Levings MK, Sangregorio R, Roncarolo MG (2001). Human cd25(+)cd4(+) t regulatory cells suppress naive and memory T cell proliferation and can be expanded *in vitro* without loss of function. J Exp Med.

[79] Zhang L, Zhao Y (2007). The regulation of Foxp3 expression in regulatory CD4(+)CD25(+)T cells: multiple pathways on the road. J Cell Physiol.

[80] Walker MR, Kasprowicz DJ, Gersuk VH, Benard A, Van Landeghen M, Buckner JH, Ziegler SF (2003). Induction of FoxP3 and acquisition of T regulatory activity by stimulated human CD4+CD25- T cells. J Clin Invest.

[81] Morgan ME, van Bilsen JH, Bakker AM, Heemskerk B, Schilham MW, Hartgers FC, Elferink BG, van der Zanden L, de Vries RR, Huizinga TW, Ottenhoff TH, Toes RE (2005). Expression of FOXP3 mRNA is not confined to CD4+CD25+ T regulatory cells in humans. Hum Immunol.

[82] Allan SE, Passerini L, Bacchetta R, Crellin N, Dai M, Orban PC, Ziegler SF, Roncarolo MG, Levings MK (2005). The role of 2 FOXP3 isoforms in the generation of human CD4+ Tregs. J Clin Invest.

[83] Tiemessen MM, Mitchell TJ, Hendry L, Whittaker SJ, Taams LS, John S (2006). Lack of suppressive CD4+CD25+FOXP3+ T cells in advanced stages of primary cutaneous T-cell lymphoma. J Invest Dermatol.

[84] Yang ZZ, Novak AJ, Ziesmer SC, Witzig TE, Ansell SM (2007). CD70+ non-Hodgkin lymphoma B cells induce Foxp3 expression and regulatory function in intratumoral CD4+CD25 T cells. Blood.

[85] Wing JB, Kitagawa Y, Locci M, Hume H, Tay C, Morita T, Kidani Y, Matsuda K, Inoue T, Kurosaki T, Crotty S, Coban C, Ohkura N (2017). A distinct subpopulation of CD25- T-follicular regulatory cells localizes in the germinal centers. Proc Natl Acad Sci U S A.

[86] Zhu Y, Zou L, Liu YC (2015). T follicular helper cells, T follicular regulatory cells and autoimmunity. Int Immunol.

[87] Ma CS, Phan TG (2017). Here, there and everywhere: T follicular helper cells on the move. Immunology.

[88] Belanger S, Crotty S (2016). Dances with cytokines, featuring TFH cells, IL-21, IL-4 and B cells. Nat Immunol.

[89] Eto D, Lao C, DiToro D, Barnett B, Escobar TC, Kageyama R, Yusuf I, Crotty S (2011). IL-21 and IL-6 are critical for different aspects of B cell immunity and redundantly induce optimal follicular helper CD4 T cell (Tfh) differentiation. PLoS One.

[90] Ma CS, Deenick EK (2014). Human T follicular helper (Tfh) cells and disease. Immunol Cell Biol.

[91] Bindea G, Mlecnik B, Tosolini M, Kirilovsky A, Waldner M, Obenauf AC, Angell H, Fredriksen T, Lafontaine L, Berger A, Bruneval P, Fridman WH, Becker C (2013). Spatiotemporal dynamics of intratumoral immune cells reveal the immune landscape in human cancer. Immunity.

[92] Cha Z, Zang Y, Guo H, Rechlic JR, Olasnova LM, Gu H, Tu X, Song H, Qian B (2013). Association of peripheral CD4+ CXCR5+ T cells with chronic lymphocytic leukemia. Tumour Biol.

[93] Gu-Trantien C, Loi S, Garaud S, Equeter C, Libin M, de Wind A, Ravoet M, Le Buanec H, Sibille C, Manfouo-Foutsop G, Veys I, Haibe-Kains B, Singhal SK (2013). CD4(+) follicular helper T cell infiltration predicts breast cancer survival. J Clin Invest.

[94] Jia Y, Zeng Z, Li Y, Li Z, Jin L, Zhang Z, Wang L, Wang FS (2015). Impaired function of CD4+ T follicular helper (Tfh) cells associated with hepatocellular carcinoma progression. PLoS One.

[95] Shi W, Li X, Cha Z, Sun S, Wang L, Jiao S, Yang B, Shi Y, Wang Z, Wu Z, Dai G (2014). Dysregulation of circulating follicular helper T cells in nonsmall cell lung cancer. DNA Cell Biol.

[96] Zhou DM, Xu YX, Zhang LY, Sun Y, Wang ZY, Yuan YQ, Fu JX (2017). The role of follicular T helper cells in patients with malignant lymphoid disease. Hematology.

[97] Myklebust JH, Irish JM, Brody J, Czerwinski DK, Houot R, Kohrt HE, Timmerman J, Said J, Green MR, Delabie J, Kolstad A, Alizadeh AA, Levy R (2013). High PD-1 expression and suppressed cytokine signaling distinguish T cells infiltrating follicular lymphoma tumors from peripheral T cells. Blood.

[98] Yang ZZ, Grote DM, Ziesmer SC, Xiu B, Novak AJ, Ansell SM (2015). PD-1 expression defines two distinct T-cell sub-populations in follicular lymphoma that differentially impact patient survival. Blood Cancer J.

[99] Travert M, Ame-Thomas P, Pangault C, Morizot A, Micheau O, Semana G, Lamy T, Fest T, Tarte K, Guillaudeux T (2008). CD40 ligand protects from TRAIL-induced apoptosis in follicular lymphomas through NF-kappaB activation and up-regulation of c-FLIP and Bcl-xL. J Immunol.

[100] Sage PT, Sharpe AH (2016). T follicular regulatory cells. Immunol Rev.

[101] Chung Y, Tanaka S, Chu F, Nurieva RI, Martinez GJ, Rawal S, Wang YH, Lim H, Reynolds JM, Zhou XH, Fan HM, Liu ZM, Neelapu SS (2011). Follicular regulatory T cells expressing Foxp3 and Bcl-6 suppress germinal center reactions. Nat Med.

[102] Linterman MA, Pierson W, Lee SK, Kallies A, Kawamoto S, Rayner TF, Srivastava M, Divekar DP, Beaton L, Hogan JJ, Fagarasan S, Liston A, Smith KG (2011). Foxp3+ follicular regulatory T cells control the germinal center response. Nat Med.

[103] Wollenberg I, Agua-Doce A, Hernandez A, Almeida C, Oliveira VG, Faro J, Graca L (2011). Regulation of the germinal center reaction by Foxp3+ follicular regulatory T cells. J Immunol.

[104] Rajnai H, Bodor C, Balogh Z, Gagyi E, Csomor J, Krenacs T, Toth E, Matolcsy A (2012). Impact of the reactive microenvironment on the bone marrow involvement of follicular lymphoma. Histopathology.

[105] Nedelkovska H, Rosenberg AF, Hilchey SP, Hyrien O, Burack WR, Quataert SA, Baker CM, Azadniv M, Welle SL, Ansell SM, Kim M, Bernstein SH (2016). Follicular lymphoma Tregs have a distinct transcription profile impacting their migration and retention in the malignant lymph node. PLoS One.

[106] Lim HW, Hillsamer P, Kim CH (2004). Regulatory T cells can migrate to follicles upon T cell activation and suppress GC-Th cells and GC-Th cell-driven B cell responses. J Clin Invest.

[107] Sage PT, Francisco LM, Carman CV, Sharpe AH (2013). The receptor PD-1 controls follicular regulatory T cells in the lymph nodes and blood. Nat Immunol.

[108] Wing JB, Ise W, Kurosaki T, Sakaguchi S (2014). Regulatory T cells control antigen-specific expansion of Tfh cell number and humoral immune responses via the coreceptor CTLA-4. Immunity.

[109] Sage PT, Paterson AM, Lovitch SB, Sharpe AH (2014). The coinhibitory receptor CTLA-4 controls B cell responses by modulating T follicular helper, T follicular regulatory, and T regulatory cells. Immunity.

[110] Schneider H, Downey J, Smith A, Zinselmeyer BH, Rush C, Brewer JM, Wei B, Hogg N, Garside P, Rudd CE (2006). Reversal of the TCR stop signal by CTLA-4. Science.

[111] Moon HG, Tae YM, Kim YS, Gyu Jeon S, Oh SY, Song Gho Y, Zhu Z, Kim YK (2010). Conversion of Th17-type into Th2-type inflammation by acetyl salicylic acid via the adenosine and uric acid pathway in the lung. Allergy.

[112] Ivanova EA, Orekhov AN (2015). T helper lymphocyte subsets and plasticity in autoimmunity and cancer: an overview. Biomed Res Int.

[113] Sahoo A, Wali S, Nurieva R (2016). T helper 2 and T follicular helper cells: regulation and function of interleukin-4. Cytokine Growth Factor Rev.

[114] Vijayanand P, Seumois G, Simpson LJ, Abdul-Wajid S, Baumjohann D, Panduro M, Huang X, Interlandi J, Djuretic IM, Brown DR, Sharpe AH, Rao A, Ansel KM (2012). Interleukin-4 production by follicular helper T cells requires the conserved Il4 enhancer hypersensitivity site V. Immunity.

[115] Harada Y, Tanaka S, Motomura Y, Ohno S, Yanagi Y, Inoue H, Kubo M (2012). The 3’ enhancer CNS2 is a critical regulator of interleukin-4-mediated humoral immunity in follicular helper T cells. Immunity.

[116] Zaretsky AG, Taylor JJ, King IL, Marshall FA, Mohrs M, Pearce EJ (2009). T follicular helper cells differentiate from Th2 cells in response to helminth antigens. J Exp Med.

[117] Gocheva V, Wang HW, Gadea BB, Shree T, Hunter KE, Garfall AL, Berman T, Joyce JA (2010). IL-4 induces cathepsin protease activity in tumor-associated macrophages to promote cancer growth and invasion. Genes Dev.

[118] Harris J, De Haro SA, Master SS, Keane J, Roberts EA, Delgado M, Deretic V (2007). T helper 2 cytokines inhibit autophagic control of intracellular Mycobacterium tuberculosis. Immunity.

[119] Wynn TA (2015). Type 2 cytokines: mechanisms and therapeutic strategies. Nat Rev Immunol.

[120] Nakayamada S, Kanno Y, Takahashi H, Jankovic D, Lu KT, Johnson TA, Sun HW, Vahedi G, Hakim O, Handon R, Schwartzberg PL, Hager GL, O’Shea JJ (2011). Early Th1 cell differentiation is marked by a Tfh cell-like transition. Immunity.

[121] de Wit J, Jorritsma T, Makuch M, Remmerswaal EB, Klaasse Bos H, Souwer Y, Neefjes J, ten Berge IJ, van Ham SM (2014). Human B cells promote T-cell plasticity to optimize antibody response by inducing coexpression of T(H)1/T(FH) signatures. J Allergy Clin Immunol.

[122] Ballesteros-Tato A, Randall TD, Lund FE, Spolski R, Leonard WJ, Leon B (2016). T follicular helper cell plasticity shapes pathogenic T helper 2 cell-mediated immunity to inhaled house dust mite. Immunity.

[123] Cannons JL, Lu KT, Schwartzberg PL (2013). T follicular helper cell diversity and plasticity. Trends Immunol.

[124] Lu KT, Kanno Y, Cannons JL, Handon R, Bible P, Elkahloun AG, Anderson SM, Wei L, Sun H, O’Shea JJ, Schwartzberg PL (2011). Functional and epigenetic studies reveal multistep differentiation and plasticity of in vitro-generated and in vivo-derived follicular T helper cells. Immunity.

[125] Zhang X, Zhang Y, Liu X, Fang A, Wang J, Yang Y, Wang L, Du L, Wang C (2016). Direct quantitative detection for cell-free miR-155 in urine: a potential role in diagnosis and prognosis for non-muscle invasive bladder cancer. Oncotarget.

[126] Qiu H, Wu H, Chan V, Lau CS, Lu Q (2017). Transcriptional and epigenetic regulation of follicular T-helper cells and their role in autoimmunity. Autoimmunity.

[127] Wan YY, Flavell RA (2007). Regulatory T-cell functions are subverted and converted owing to attenuated Foxp3 expression. Nature.

[128] Tsuji M, Komatsu N, Kawamoto S, Suzuki K, Kanagawa O, Honjo T, Hori S, Fagarasan S (2009). Preferential generation of follicular B helper T cells from Foxp3+ T cells in gut Peyer’s patches. Science.

[129] Koch MA, Tucker-Heard G, Perdue NR, Killebrew JR, Urdahl KB, Campbell DJ (2009). The transcription factor T-bet controls regulatory T cell homeostasis and function during type 1 inflammation. Nat Immunol.

[130] Dominguez-Villar M, Baecher-Allan CM, Hafler DA (2011). Identification of T helper type 1-like, Foxp3+ regulatory T cells in human autoimmune disease. Nat Med.

[131] Oldenhove G, Bouladoux N, Wohlfert EA, Hall JA, Chou D, Dos Santos L, O’Brien S, Blank R, Lamb E, Natarajan S, Kastenmayer R, Hunter C, Grigg ME (2009). Decrease of Foxp3+ Treg cell number and acquisition of effector cell phenotype during lethal infection. Immunity.

[132] Stock P, Akbari O, Berry G, Freeman GJ, Dekruyff RH, Umetsu DT (2004). Induction of T helper type 1-like regulatory cells that express Foxp3 and protect against airway hyper-reactivity. Nat Immunol.

[133] Ayyoub M, Deknuydt F, Raimbaud I, Dousset C, Leveque L, Bioley G, Valmori D (2009). Human memory FOXP3+ Tregs secrete IL-17 ex vivo and constitutively express the T(H)17 lineage-specific transcription factor RORgamma t. Proc Natl Acad Sci U S A.

[134] Deknuydt F, Bioley G, Valmori D, Ayyoub M (2009). IL-1beta and IL-2 convert human Treg into T(H)17 cells. Clin Immunol.

[135] Xu L, Kitani A, Fuss I, Strober W (2007). Cutting edge: regulatory T cells induce CD4+CD25-Foxp3- T cells or are self-induced to become Th17 cells in the absence of exogenous TGF-beta. J Immunol.

[136] Peters JH, Hilbrands LB, Koenen HJ, Joosten I (2008). Ex vivo generation of human alloantigen-specific regulatory T cells from CD4(pos)CD25(high) T cells for immunotherapy. PLoS One.

[137] Miller SA, Weinmann AS (2010). Molecular mechanisms by which T-bet regulates T-helper cell commitment. Immunol Rev.

[138] Ansel KM, Djuretic I, Tanasa B, Rao A (2006). Regulation of Th2 differentiation and Il4 locus accessibility. Annu Rev Immunol.

[139] Zheng Y, Josefowicz SZ, Kas A, Chu TT, Gavin MA, Rudensky AY (2007). Genome-wide analysis of Foxp3 target genes in developing and mature regulatory T cells. Nature.

[140] Hirahara K, Vahedi G, Ghoreschi K, Yang XP, Nakayamada S, Kanno Y, O’Shea JJ, Laurence A (2011). Helper T-cell differentiation and plasticity: insights from epigenetics. Immunology.

[141] Murphy KM, Stockinger B (2010). Effector T cell plasticity: flexibility in the face of changing circumstances. Nat Immunol.

[142] Bao Y, Cao X (2016). Epigenetic control of B cell development and B-cell-related immune disorders. Clin Rev Allergy Immunol.

[143] Kitagawa Y, Ohkura N, Sakaguchi S (2015). Epigenetic control of thymic Treg-cell development. Eur J Immunol.

[144] Kondilis-Mangum HD, Wade PA (2013). Epigenetics and the adaptive immune response. Mol Aspects Med.

[145] Lin Q, Chauvistre H, Costa IG, Gusmao EG, Mitzka S, Hanzelmann S, Baying B, Klisch T, Moriggl R, Hennuy B, Smeets H, Hoffmann K, Benes V (2015). Epigenetic program and transcription factor circuitry of dendritic cell development. Nucleic Acids Res.

[146] Mukasa R, Balasubramani A, Lee YK, Whitley SK, Weaver BT, Shibata Y, Crawford GE, Hatton RD, Weaver CT (2010). Epigenetic instability of cytokine and transcription factor gene loci underlies plasticity of the T helper 17 cell lineage. Immunity.

[147] Nagata DE, Ting HA, Cavassani KA, Schaller MA, Mukherjee S, Ptaschinski C, Kunkel SL, Lukacs NW (2015). Epigenetic control of Foxp3 by SMYD3 H3K4 histone methyltransferase controls iTreg development and regulates pathogenic T-cell responses during pulmonary viral infection. Mucosal Immunol.

[148] Sellars M, Huh JR, Day K, Issuree PD, Galan C, Gobeil S, Absher D, Green MR, Littman DR (2015). Regulation of DNA methylation dictates Cd4 expression during the development of helper and cytotoxic T cell lineages. Nat Immunol.

[149] Wu H, Zhao M, Yoshimura A, Chang C, Lu Q (2016). Critical link between epigenetics and transcription factors in the induction of autoimmunity: a comprehensive review. Clin Rev Allergy Immunol.

[150] Bernstein BE, Mikkelsen TS, Xie X, Kamal M, Huebert DJ, Cuff J, Fry B, Meissner A, Wernig M, Plath K, Jaenisch R, Wagschal A, Feil R (2006). A bivalent chromatin structure marks key developmental genes in embryonic stem cells. Cell.

[151] Wei G, Wei L, Zhu J, Zang C, Hu-Li J, Yao Z, Cui K, Kanno Y, Roh TY, Watford WT, Schones DE, Peng W, Sun HW (2009). Global mapping of H3K4me3 and H3K27me3 reveals specificity and plasticity in lineage fate determination of differentiating CD4+ T cells. Immunity.

[152] Oestreich KJ, Weinmann AS (2012). Encoding stability versus flexibility: lessons learned from examining epigenetics in T helper cell differentiation. Curr Top Microbiol Immunol.

[153] Cook KD, Shpargel KB, Starmer J, Whitfield-Larry F, Conley B, Allard DE, Rager JE, Fry RC, Davenport ML, Magnuson T, Whitmire JK, Su MA (2015). T follicular helper cell-dependent clearance of a persistent virus infection requires T cell expression of the histone demethylase UTX. Immunity.

[154] Rezvani K, de Lavallade H (2011). Vaccination strategies in lymphomas and leukaemias: recent progress. Drugs.

[155] Cioc AM, Vanderwerf SM, Peterson BA, Robu VG, Forster CL, Pambuccian SE (2008). Rituximab-induced changes in hematolymphoid tissues found at autopsy. Am J Clin Pathol.

[156] Schroder C, Azimzadeh AM, Wu G, Price JO, Atkinson JB, Pierson RN (2003). Anti-CD20 treatment depletes B-cells in blood and lymphatic tissue of cynomolgus monkeys. Transpl Immunol.

[157] Baumjohann D, Preite S, Reboldi A, Ronchi F, Ansel KM, Lanzavecchia A, Sallusto F (2013). Persistent antigen and germinal center B cells sustain T follicular helper cell responses and phenotype. Immunity.

[158] M’Hidi H, Thibult ML, Chetaille B, Rey F, Bouadallah R, Nicollas R, Olive D, Xerri L (2009). High expression of the inhibitory receptor BTLA in T-follicular helper cells and in B-cell small lymphocytic lymphoma/chronic lymphocytic leukemia. Am J Clin Pathol.

[159] Aydar Y, Sukumar S, Szakal AK, Tew JG (2005). The influence of immune complex-bearing follicular dendritic cells on the IgM response, Ig class switching, and production of high affinity IgG. J Immunol.

[160] Gaspal FM, McConnell FM, Kim MY, Gray D, Kosco-Vilbois MH, Raykundalia CR, Botto M, Lane PJ (2006). The generation of thymus-independent germinal centers depends on CD40 but not on CD154, the T cell-derived CD40-ligand. Eur J Immunol.

[161] Estes JD, Thacker TC, Hampton DL, Kell SA, Keele BF, Palenske EA, Druey KM, Burton GF (2004). Follicular dendritic cell regulation of CXCR4-mediated germinal center CD4 T cell migration. J Immunol.

[162] Kim J, Kim DW, Chang W, Choe J, Kim J, Park CS, Song K, Lee I (2012). Wnt5a is secreted by follicular dendritic cells to protect germinal center B cells via Wnt/Ca2+/NFAT/NF-kappaB-B cell lymphoma 6 signaling. J Immunol.

[163] Lee SK, Rigby RJ, Zotos D, Tsai LM, Kawamoto S, Marshall JL, Ramiscal RR, Chan TD, Gatto D, Brink R, Yu D, Fagarasan S, Tarlinton DM (2011). B cell priming for extrafollicular antibody responses requires Bcl-6 expression by T cells. J Exp Med.

[164] Ok CY, Singh RR, Vega F (2012). Aberrant activation of the hedgehog signaling pathway in malignant hematological neoplasms. Am J Pathol.

